# Studying serum neurofilament light chain levels as a potential new biomarker for small fiber neuropathy

**DOI:** 10.1111/ene.16192

**Published:** 2024-01-08

**Authors:** Panoraia Baka, Livia Steenken, Fabiola Escolano‐Lozano, Falk Steffen, Aikaterini Papagianni, Claudia Sommer, Esther Pogatzki‐Zahn, Silke Hirsch, Maria Protopapa, Stefan Bittner, Frank Birklein

**Affiliations:** ^1^ Department of Neurology University Medical Center of the Johannes Gutenberg University Mainz Mainz Germany; ^2^ Department of Neurology University Hospital of Würzburg Würzburg Germany; ^3^ Department of Anaesthesiology, Intensive Care and Pain Medicine University Hospital Münster Münster Germany

**Keywords:** axonal degeneration, biomarker, intraepidermal nerve fiber density, serum neurofilament light chain, small fiber neuropathy

## Abstract

**Background and purpose:**

Diagnosing small fiber neuropathies can be challenging. To address this issue, whether serum neurofilament light chain (sNfL) could serve as a potential biomarker of damage to epidermal Aδ‐ and C‐fibers was tested.

**Methods:**

Serum NfL levels were assessed in 30 patients diagnosed with small fiber neuropathy and were compared to a control group of 19 healthy individuals. Electrophysiological studies, quantitative sensory testing and quantification of intraepidermal nerve fiber density after skin biopsy were performed in both the proximal and distal leg.

**Results:**

Serum NfL levels were not increased in patients with small fiber neuropathy compared to healthy controls (9.1 ± 3.9 and 9.4 ± 3.8, *p* = 0.83) and did not correlate with intraepidermal nerve fiber density at the lateral calf or lateral thigh or with other parameters of small fiber impairment.

**Conclusion:**

Serum NfL levels cannot serve as a biomarker for small fiber damage.

## INTRODUCTION

Small fiber neuropathy (SFN) comprises a heterogeneous group of disorders that affect thinly myelinated Aδ‐fibers and unmyelinated C‐fibers [1]. SFN is reported to be associated with a wide range of diseases or conditions [2, 3, 4], although few of these have a causal relationship to SFN like diabetes and impaired glucose tolerance [5, 6]. In the majority of patients with SFN, particularly the elderly, no specific etiology can be identified [7, 8]. The exact pathophysiology, which leads to the particular damage of small nerve fiber endings, is still unclear. It is also unclear whether nerve fiber degeneration occurs only at the very distal endings or also involves the neuron in the dorsal root ganglion [9, 10, 11].

The diagnosis of SFN is based on the presence of neuropathic pain and dysesthesia, along with normal nerve conduction and decreased warm and cold sensation on quantitative sensory testing (QST), as well as reduced intraepidermal nerve fiber density (IENFD) at the lateral calf [1, 4, 12]. SFN typically presents as a distally symmetric “dying‐back” pattern in most patients, but in some cases it can manifest with a patchy and asymmetric pattern, making it challenging to diagnose and differentiate from functional pain disorders without structural nerve damage [13, 14]. To overcome these diagnostic difficulties, but also in order to get an insight into the pathophysiology of SFN, it was investigated whether thin nerve fiber degeneration in SFN can increase serum neurofilament light chain (sNfL) levels, thereby making it a potential biomarker.

Neurofilaments confer structural stability and are present in dendrites and neuronal soma, as well as in axons, where their expression is particularly high [15, 16, 17]. NfL is consistently released at lower levels from axons, probably in an age‐dependent fashion, with elevated NfL release observed in older individuals [17]. Importantly, NfL is also found to be correlated with neuronal damage in the cerebrospinal fluid (CSF) and serum of patients with conditions such as traumatic brain injury [18], neurodegenerative diseases like amyotrophic lateral sclerosis [19, 20] and chronic inflammatory conditions like multiple sclerosis [21, 22, 23]. Notably, there is a growing body of evidence indicating that blood NfL levels are elevated in polyneuropathies [24, 25, 26, 27, 28, 29, 30, 31, 32, 33] and correlate with neuropathy acuteness and progression [26, 32, 34, 35, 36]. As there is damage/degeneration of small fiber axons [37, 38, 39, 40] and neurofilaments are important components of the axon, it was hypothesized that NfL, which is broadly investigated and significantly increased in other polyneuropathies, could be a surrogate for fiber loss in SFN.

## MATERIALS AND METHODS

### Study design and patients

A cross‐sectional observational study was conducted and included patients with SFN referred to the Neurology Department at the University Medical Center of the Johannes Gutenberg University Mainz (Mainz, Germany), from March 2019 to December 2020. The study was conducted according to the Declaration of Helsinki and was approved by the Ethics Committee of the Rhineland‐Palatinate Medical Association (837.437.17). A written informed consent was obtained from each participant.

During their first visit, patients received a comprehensive examination by an experienced neurologist. Patients diagnosed with SFN of any etiology were included. The diagnosis SFN was made according to guidelines and landmark papers [1, 41]. More specifically, only patients who met the Diabetic Neuropathy Study Group of the European Association for the Study of Diabetes (NEURODIAB) criteria [41, 42] for a definite SFN diagnosis, which means clinical evidence of small fiber damage, normal sural nerve conduction studies (NCS), abnormal thermal thresholds in QST at the foot and/or reduced IENFD at the ankle were included.

Age‐ and sex‐matched healthy controls (HCs) without any known acute (at the time of recruitment) or chronic disease and in particular absence of any signs of neuropathy were also investigated. Due to the positive correlation between age and sNfL [43], it was sought to create homogeneous age‐matched study groups. Participants (both patients with SFN and HCs) with comorbidities that could affect sNfL (e.g., other neurological disorders, malignancies) were excluded.

### Toronto Clinical Neuropathy Scale and symptom evaluation

The Toronto Clinical Neuropathy Scale (TCNS) [44], which assesses symptoms and objective sensory‐motor signs, was used as a clinical tool to define neuropathy severity. A TCNS score from 0 to 5 represents no/very mild neuropathy, 6–8 mild neuropathy, 9–11 moderate neuropathy, and a score ≥12 represents severe neuropathy [44]. Pain intensity between 1 and 3 on the 11‐point numerical rating scale was regarded as mild, 4–6 as moderate and 7–10 as severe pain, in accordance with previous publications [45, 46, 47].

### Serum NfL measurement

Serum NfL levels were measured using the same protocol described previously [22]. In brief, whole blood was collected from all patients and controls in 7.5 mL S‐Monovette® Serum Gel (Sarstedt, Germany). Blood was allowed to clot for about 5 min after sampling. Next, samples were spun at 1400 ***g*** at room temperature for 10 min. Directly after centrifugation, the serum was evenly transferred (1 mL/tube) to 1 mL polypropylene tubes and locally stored at −80°C. sNfL was measured in several rounds by SiMoA HD‐1 (Quanterix, USA) using the NF‐Light Advantage Kit (Quanterix) from the same batch according to the manufacturer's instructions. Resorufin‐β‐d‐galactopyranoside was incubated at 33°C for 60 min prior to running the assay. Samples were measured in duplicate. The coefficient of variation (as a percentage) of each sample was obtained by dividing the standard deviation of both replicates by the mean of both replicates multiplied by 100. sNfL measurements were performed in a blinded fashion without information on clinical data.

### Electrophysiological studies

All patients and controls underwent NCS. Ulnar and tibial motor nerve conduction velocity (NCV), compound muscle action potential and antidromic ulnar and sural sensory NCV and sensory nerve action potential (SNAP) were performed (right side of the body, or predominant side of symptoms) under controlled conditions using standard methods [48].

### Quantitative sensory testing

Quantitative sensory testing (QST) was performed according to the established protocol of the German Research Network on Neuropathic Pain (DFNS) [49] at the lateral calf (test area) and ipsilateral cheek (control area). Thermal and mechanical detection and pain thresholds, paradoxical heat sensations with alternating thermal stimuli, dynamic mechanical allodynia, wind‐up ratio for painful pinprick stimuli and the vibration disappearance threshold were determined. Since 5% of healthy individuals can present with at least one pathological test [49], determination of pathological QST required the presence of at least two pathological tests, with the exception of the presence of paradoxical heat sensations or dynamic mechanical allodynia, since they do not normally occur in healthy humans. Reference values are according to the DFNS [49].

### Skin biopsies

According to the consensus paper by the European Federation of Neurological Societies [1], skin punch biopsies were obtained 10 cm proximal to lateral malleolus and 20 cm distal to spina iliaca with a disposable 6‐mm punch biopsy after subcutaneous local anesthesia. All skin samples were processed to estimate IENFD according to a previously published protocol [50]. Skin biopsies were gathered and sent to the histology laboratory of the Department of Neurology, University of Würzburg, Germany. The IENFD was determined following standardized counting rules [1] by an investigator blinded to subject allocation. Results were then evaluated by an experienced clinician also blinded to the subject.

### Statistical analysis

Statistical analyses were performed using IBM SPSS 23 Statistics, version 23.0, and GraphPad Prism 9 for Windows. The level of statistical significance was set at *p* < 0.05. All data were tested for normal distribution by the D'Agostino−Pearson test and by visual inspection of the distribution. Comparison of data between groups was performed with *t* tests. Pearson correlation analyses were performed to explore associations between sNfL levels and clinical and histological parameters, as well as neurophysiological data. Categorical data were analyzed with *χ* ^2^ tests.

Regarding QST data, raw data were transformed into *z* values as previously described [51] allowing comparison between different sexes and ages. For individual assessments, values are regarded as pathological if the individual results at the test area lie outside the 95% confidence interval (CI) of the age‐adapted reference values [51].

## RESULTS

### Patients' characteristics

This study included 30 patients with SFN (*n* = 30, 11 female and 19 male, mean age ± SD 46 ± 11 years), in most cases idiopathic (81.8%) (Figure S1), and 19 HCs (*n* = 19, 10 female and nine male, mean age ± SD 46 ± 8 years). There were no differences in age or sex between the two groups (Table 1). The median duration of the SFN was 18 months (95% CI 24.5–60.1 months). The median TCNS score was 8 (95% CI 6.5–9.3) representing a mild neuropathy [44]. The median pain intensity the week before recruitment, which was rated on an 11‐point numerical rating scale (NRS 0–10) was 5 (95% CI 4.2–5.8), the median pain intensity on the examination day was 3 (95% CI 2.6–4.6) and the median strongest pain intensity in the week before recruitment was 8 (95% CI 6.2–7.6).

**TABLE 1:** Summary of clinical characteristics and results of NCS of the study population.

	SFN *n* = 30	Healthy controls *n* = 19	*p* value
Age	46 ± 11	46 ± 8	0.88
Sex (W/M)	11/19	10/9	0.54
Sural SNAP (μV)	11.7 ± 4.6	10 ± 3.5	0.17
Sural sensCV (m/s)	52.8 ± 10.7	54.1 ± 9	0.68
Tibial CMAP (mV)	17.7 ± 6.1	19.4 ± 10.1	0.52
Tibial motorCV (m/s)	50 ± 6.6	59.7 ± 9	0.14
Ulnar CMAP (mV)	15 ± 3.8	13.8 ± 3.1	0.24
Ulnar motorCV (m/s)	61.9 ± 9.6	66.5 ± 12.3	0.16
Ulnar SNAP (μV)	27.3 ± 14.5	23.1 ± 11.7	0.29
Ulnar sensCV (m/s)	58.8 ± 7.2	62.3 ± 10.6	0.18
Sural/ulnar SNAP ratio	0.55 ± 0.31	0.65 ± 0.47	0.34

Abbreviations: CMAP, compound muscle action potential; motorCV, motor nerve conduction velocity; NCS, nerve conduction studies; sensCV, sensory nerve conduction velocity; SFN, small fiber neuropathy; SNAP, sensory nerve action potential.

All recordings from NCS of both patients and the control group were within the normal limits of age‐controlled normative values. NCS of the sural nerve did not differ between patients and controls (SFN patients, mean ± SD, sural SNAP 11.7 ± 4.6, sural NCV 52.8 ± 10.7; HCs, sural SNAP 10 ± 3.5, sural NCV 54.1 ± 9.1; *p* = 0.17 and *p* = 0.68 respectively). Large fiber neuropathy was not detected in any of the included patients. The sural/ulnar SNAP ratio, which is discussed as an index of early length‐dependent polyneuropathy [52, 53, 54], was additionally calculated, and no differences were found between patients (mean ± SD 0.55 ± 0.31) and HCs (0.65 ± 0.47) (*t* test, *p* = 0.34).

### Assessment of the small fibers and diagnosis of SFN

**FIGURE 1:**
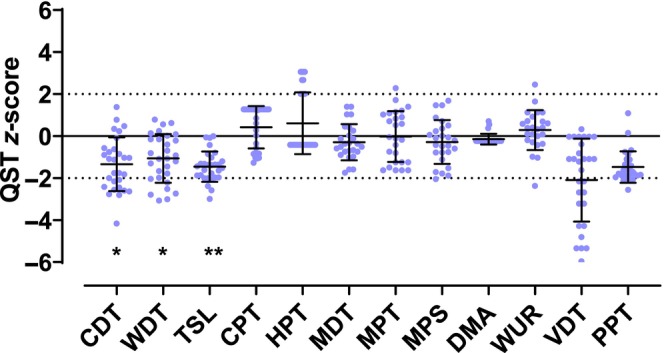
**Quantitative sensory testing of all patients**. QST *z* scores of the SFN patients (mean/SD at the lateral calf). The zero line denotes the mean value of the control group, the dashed lines in each case 1.96 times the standard deviation from the mean value of the control group, i.e., the range in which the values of the control group lie within a 95% probability (95% confidence interval). Negative *z* scores denote loss of sensitivity. The asterisks denote the significant differences of the raw data between patients and control group: CDT *p* < 0.001, WDT *p* < 0.001, TSL *p* < 0.00, VDT *p* = 0.033 (*p < 0.05; **p < 0.001). CDT, cold detection threshold; CPT, cold pain threshold; DMA, dynamical mechanical allodynia; HPT, heat pain threshold; MDT, mechanical detection threshold; MPS, mechanical pain sensitivity; MPT, mechanical pain threshold; PPT, pressure pain threshold; TSL, thermal sensory limen; VDT, vibration detection threshold; WDT, warm detection threshold; WUR, wind‐up ratio.

Quantitative sensory testing (QST) revealed predominant loss of thermal perception, that is, warm detection threshold (WDT), cold detection threshold (CDT) and thermal sensory limen (TSL) (Figure 1). Individually, 11/29 patients with SFN were below the normal limits (−1.96 standard deviations) of CDT, 8/29 of WDT and 8/29 of TSL. QST data from one patient are missing because of no‐show at the QST test site.

**FIGURE 2:**
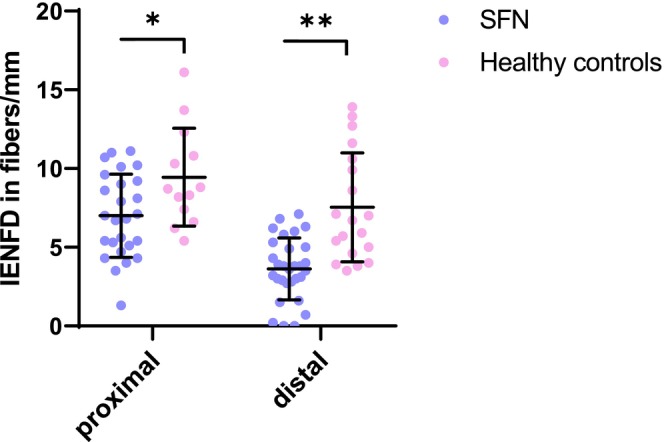
**Comparing intraepidermal nerve fiber density between patients and healthy controls**. Intraepidermal nerve fiber density (fibers/mm). Horizontal lines indicate the mean and the bars the standard deviation (SD) of patients and healthy controls. Patients have significantly fewer fibers/mm at both the proximal (*p* = 0.014) and distal (*p* < 0.01) biopsy sites compared to controls. The number of proximal skin biopsies of HCs was smaller (*n* = 13) because not all consented to a second skin biopsy. HC, healthy controls; IENFD, intraepidermal nerve fiber density; SFN, small fiber neuropathy.

**FIGURE 3:**
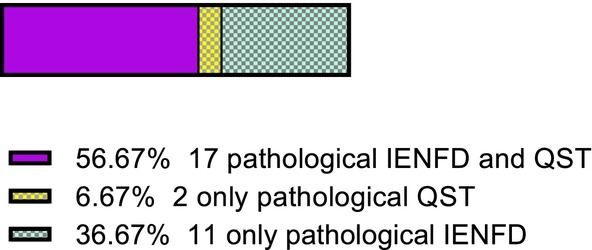
**Defining small fiber polyneuropathy patients according to the NEURODIAB criteria**. Diagnosis of definite SFN based on NEURODIAB criteria. All 30 patients fulfilled the criteria for definite SFN, 17 of whom had both pathological IENFD and abnormal quantitative sensory testing (QST) thermal thresholds at the foot, two had only pathological thermal threshold abnormalities and 11 had only pathological IENFD. All patients had clinical evidence of small fiber damage and normal sural nerve conduction studies. IENFD, intraepidermal nerve fiber density; SFN, small fiber neuropathy.

Both the proximal IENFD (patients, 7 ± 2.6 fibers/mm; HCs, 9.5 ± 3.1 fibers/mm; *p* = 0.014) and distal IENFD (patients, 3.6 ± 2 fibers/mm; HCs, 7.5 ± 3.5 fibers/mm; *p* < 0.01) were lower in SFN patients (Figure 2). 25/30 SFN patients had a pathological low distal IENFD but also 2/19 HCs presented with an asymptomatic low IENFD, based on age‐ and sex‐adjusted normative values [1, 55]. All patients fulfilled the criteria for a definite SFN [41]; 17 had both pathological IENFD and abnormal QST thermal thresholds (Figure 3).

### Serum NfL in patients with SFN

**FIGURE 4:**
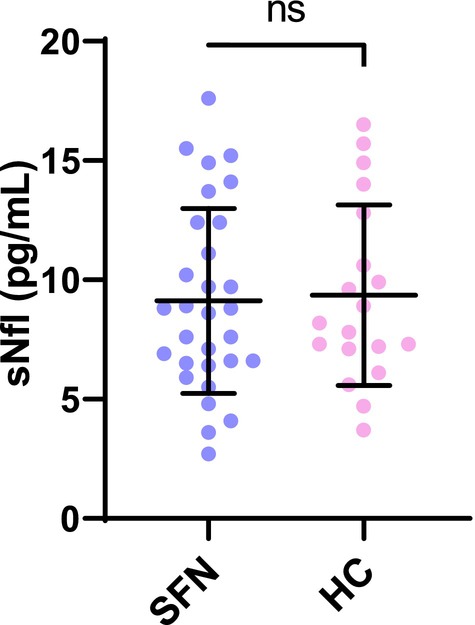
**Comparing serum Neurofilament between patients and healthy controls**. sNfL concentrations of 30 patients with SFN and 19 age‐matched healthy controls. Horizontal lines indicate the mean and the bars the standard deviation (SD) of patients and healthy controls. No difference was detected between the concentrations of SFN patients (mean ± SD, 9.1 ± 3.9) and healthy controls (9.4 ± 3.8), *p* = 0.83. HC, healthy controls; ns, non‐significant; SFN, small fiber neuropathy; sNfl, serum neurofilament light chain.

Serum NfL could be measured in all participants; levels did not differ (*p* = 0.83) between patients with SFN (9.1 ± 3.9) and HCs (9.4 ± 3.8). The distribution of individual results broadly overlaps (Figure 4). Although mean age was similar in the two groups, the correlation of age and sNfL was calculated separately for the two groups and they both revealed a strong positive Pearson correlation (for HCs *p* = 0.001, *r* = 0.70; for patients with SFN *p* < 0.001, *r* = 0.69).

In the final step of our analysis, whether there was a correlation between sNfL and tests for small fiber integrity or a sensitive parameter for large fiber impairment, namely sural NCS, was examined (Table 2). In the SFN group, a significant correlation was found between sNfL and sural SNAP (*r* = −0.43, *p* = 0.02) although the individual values fell in the normal range for all patients. Such a correlation was not observed in HCs (sural nerve SNAP *r* = −0.09, *p* = 0.71). No correlation was observed between sNfL and sural/ulnar SNAP ratio.

**TABLE 2:** Pearson correlations between sNfL and small fiber parameters as well as sural nerve NCS.

	IENFD lateral thigh	IENFD lateral calf	CDT	WDT	MDT	MPT	TSL	VDT	Sural nerve SNAP	Sural nerve NCV	Sural/ulnar ratio
sNfL											
*r*	−0.15	−0.28	0.05	0.28	0.12	0.12	−0.03	−0.21	−0.43	−0.26	−0.17
*p*	0.46	0.14	0.81	0.14	0.56	0.56	0.89	0.28	0.02*	0.17	0.37

*Note*: There were no significant correlations between sNfL and the other results of SFN assessment in the patient cohort (*n* = 30), but there was a strong negative correlation with sural nerve SNAP.

Abbreviations: CDT, cold detection threshold; IENFD, intraepidermal nerve fiber density; MDT, mechanical detection threshold; MPT, mechanical pain threshold; NCS, nerve conduction studies; NCV, nerve conduction velocity; SFN, small fiber neuropathy; SNAP, sensory nerve action potential; sNfL, serum neurofilament light chain; TSL, thermal sensory limen; VDT, vibration disappearance threshold; WDT, warm detection threshold. *significant p value.

## DISCUSSION

Neurofilaments contribute to the growth and stability of axons in both central and peripheral neurons and maintain mitochondrial stability and microtubule content [56]. NfL is expressed in axons and is shed into the peripheral blood following axonal injury in patients with a variety of neurological diseases in the central and peripheral nervous system [57]. sNfL levels are known to be increased in peripheral sensorimotor neuropathies, particularly if they are significantly progressing [24, 25, 26, 27, 28, 29, 30, 31, 32, 33]. The availability of highly sensitive assays for NfL in serum samples [17] and the need for a quick and reproducible biomarker for nerve fiber damage particularly in clinically ambiguous cases motivated our measurements of sNfL levels in patients with pure SFN.

Our results indicate that sNfL levels of patients with SFN were not different from HCs. Furthermore, no correlation was found between any parameters reflecting small nerve fiber function and integrity and sNfL levels in our patients. That is, sNfL levels do not reflect damage to small diameter axons in SFN although ongoing axonal degeneration and regeneration might happen [37, 38, 39, 40]. sNfL are therefore not suited as a biomarker for SFN. Our negative results indicate that either the NfL content of small intraepidermal nerve fibers is too low [58] or the axonal damage in SFN is generally too subtle to detect altered NfL in serum samples. It has already been discussed before that CSF as well as blood NfL remains high for 2–3 months after a relapse and then drops to lower levels in multiple sclerosis patients [15, 17] and that serum levels in chronic inflammatory demyelinating polyneuropathy significantly decrease after 1 month of treatment and in remission periods [32, 34]; this could also affect the diagnostic accuracy of sNfL in patients with SFN, as there are no clear signs of relapses or the exact timing of fiber loss and degeneration.

Patients and HCs were homogeneous, especially in terms of sex and age. Age is an important positive predictor for sNfL [43, 59]. Our analysis confirmed this finding by presenting a strong correlation of sNfL with age in both study cohorts. Furthermore, an ultra‐sensitive fourth‐generation (single‐molecule array) immunoassay was used that can reliably measure blood levels of sNfL and detect subtle longitudinal changes in disease and in healthy controls [57, 60]. All values from patients and controls were above the detection limit.

One strength of our study is that our patients adhered to the definition of “pure” SFN. Large fiber neuropathy could not be detected in any of the patients, and QST profiles were typical for patients with small fiber damage [12, 61]. However, the negative correlation of sural SNAP with sNfL in the patient group, even though SNAP was in the normal range, might indicate some preclinical impairment of large sensory fibers. Obviously, this impairment is too subtle to cause an absolute increase of sNfL as has been shown in more progressive sensory‐motor neuropathies [25, 62, 63]. Indirectly, this interpretation is supported by the lack of correlation between sNfL and these parameters in HCs.

It was observed that a reduction in IENFD from the distal or proximal leg biopsy site was not sufficient to increase sNfL. This is in contrast to a recent study showing increased phosphorylated heavy chain neurofilaments (NfH) in plasma samples of patients with diabetic SFN [63]. In that study [63], patients had a significantly lower sural SNAP compared to their HCs which might be an indication for at least some sensory fiber damage leading to an increase in serum neurofilaments, as discussed in the previous paragraph. In contrast to those results [63], in our study sural SNAP did not differ between the two groups. NfL and NfH are equally increased in the serum and CSF of patients with amyotrophic lateral sclerosis [64] and are both equally stable for measurement [65], but fewer data are available on NfH [66], particularly when peripheral neuropathies are concerned. Moreover, the SiMoA method is a highly sensitive technique compared to the enzyme‐linked immunosorbent assay or the electrochemiluminescence‐based assay for CSF and serum samples [67, 68, 69]. Further investigations are needed to explore whether sNfL could differentiate between SFN of different etiologies. It is yet speculative, but axonal damage (and sNfL) in idiopathic SFN, prevalent in 81.8% of our study cohort, which could remain stable over a long period [4, 70, 71], might be different from SFN in systemic diseases like diabetes leading to sensorimotor neuropathy in due course, despite the clinical similarity.

The major limitation of our study is that the sample size is limited, precluding comparisons of different etiological SFN subgroups. Whilst the majority of our patients have idiopathic SFN, whose pathology might be confined to the epidermal nerve fibers, future investigations should include a sufficient number of subjects with SFN as a result of an ongoing (e.g., diabetes or thyroid dysfunction) or terminated systemic disease (e.g., drug‐related), in order to further address the role of sNfL as biomarkers for peripheral neuropathies.

## CONCLUSION

Even if measured with a very sensitive assay, sNfL levels are not suited to objectify or to monitor loss of small epidermal nerve fibers in SFN. Future research has to clarify whether the amount of NfL in epidermal nerve fibers is too low, whether other filament proteins like the recently investigated peripherin [72] are better suited to monitor small nerve fiber damage, or whether the pathophysiology of SFN is not related to an ongoing axonal damage.

## AUTHOR CONTRIBUTIONS

**Panoraia Baka:** Conceptualization; investigation; writing – original draft; methodology; validation; visualization; writing – review and editing; software; formal analysis; project administration; data curation. **Livia Steenken:** Writing – original draft; methodology; writing – review and editing; data curation; project administration; investigation. **Fabiola Escolano‐Lozano:** Writing – review and editing. **Falk Steffen:** Writing – review and editing; methodology. **Aikaterini Papagianni:** Writing – review and editing. **Claudia Sommer:** Writing – review and editing. **Esther Pogatzki‐Zahn:** Writing – review and editing. **Silke Hirsch:** Writing – review and editing. **Maria Protopapa:** Writing – review and editing. **Stefan Bittner:** Writing – review and editing; methodology. **Frank Birklein:** Conceptualization; investigation; funding acquisition; visualization; writing – review and editing; formal analysis; supervision; resources.

## CONFLICT OF INTEREST STATEMENT

EPZ received financial support from Grünenthal for research activities and advisory and lecture fees from Grünenthal, Novartis and Medtronic. All money went to the institution EPZ is working for. CS has received honoraria consulting for Algiax, Bayer, Merz and Omega on the subject of neuropathic pain. SB is supported by the Deutsche Forschungsgemeinschaft (DFG, SFB CRC‐TR‐128 and TRR355) and the Hermann and Lilly Schilling Foundation. The other authors declare no competing interests.

## Supporting information


Figure S1.

## Data Availability

The data that support the findings of this study are available on request from the corresponding author. The data are not publicly available due to privacy or ethical restrictions.
