# Supplementary material for: Studying serum neurofilament light chain levels as a potential new biomarker for small fiber neuropathy
Source: Eur J Neurol. 2024 Jan 8;31(4):e16192. doi: 10.1111/ene.16192 (PMC11235889; doi:10.1111/ene.16192)
Supplement: Supplementary file 1 — Figure S1. [file ENE-31-e16192-s001.docx]

# Supplementary Material

**Supplementary Figure 1** Etiology of small fiber neuropathy
